# 47. Impact of COVID-19 Pandemic on an Emergency Department Opt-out HIV Screening Program: An Interrupted Time Series Analysis

**DOI:** 10.1093/ofid/ofab466.047

**Published:** 2021-12-04

**Authors:** Jianli Niu, Paula Eckardt

**Affiliations:** 1 Memorial Healthcare System, Hollywood, Florida; 2 MHS, weston, FL

## Abstract

**Background:**

The coronavirus disease 2019 (COVID-19) pandemic has posed tremendous challenges to health care systems, including emergency department (ED) priorities and visits. We describe the impact of COVID-19 pandemic on ED-based “Opt-out” HIV testing at a public healthcare system in South Florida.

**Methods:**

The programmatic data of ED-based HIV testing from July 2018 to March 2021 at the Memorial Regional Hospital, Hollywood, Florida was retrospectively analyzed. Interrupted time series (ITS) analysis models were developed to evaluate the immediate and gradual effects of the pandemic on the monthly number of HIV tests over time, with an interruption point at March 2020.

**Results:**

45,185 HIV tests were recorded between July 2018 and March 2021. A mean of 1,745 (SD, 266) HIV tests per month before the COVID-19 pandemic (July 2018 to Feb 2020) and a mean of 791 (SD, 187) HIV tests per month during the pandemic period (March 2020 to March 2021) was seen (p< 0.0001). As shown in Table 1, there was a slight decline trend in the number of monthly HIV test before the pandemic (estimate -10.29, p=0.541). We estimated a significant decrease in monthly HIV tests (estimate -678.48, p = 0.008), whereas the slope change after the pandemic was non-significant (estimate 4.84, p = 0.891). The number of monthly HIV tests declined significantly during the early phase of the pandemic, particularly between March 2020 and September 2020 (all p< 0.05), with an estimated 48.0% decrease in the March 2020 (estimate -678.48, p = 0.007), 43% in the April 2020 (estimate -673.65, p=0.007), and 50.7% in the May 2020 (estimate -668.83, p=0.009), compared with the same month of the pre-pandemic period (Figure 1). This decline in number of monthly HIV tests is consistent with the first wave of the COVID-19 pandemic in South Florida. Number of decreased monthly HIV tests from October 2020 through March 2021 was less pronounced (all p >0.05) and returned to pre-pandemic levels.

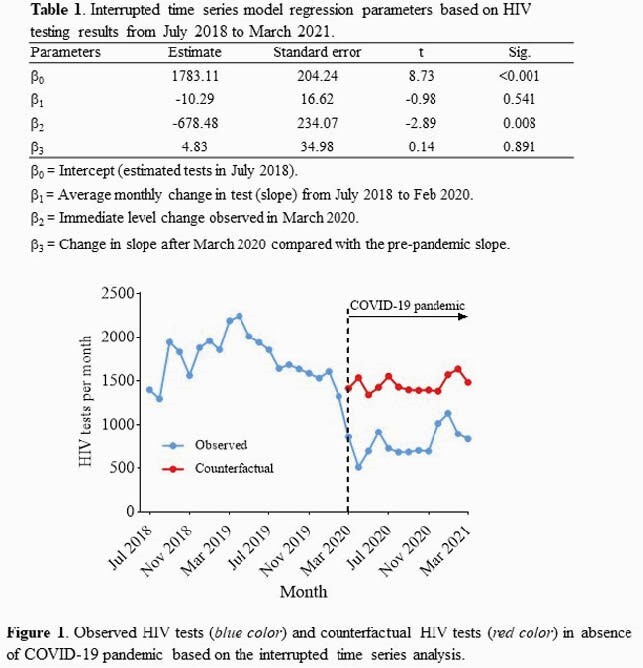

**Conclusion:**

The COVID-19 pandemic led to a significant and immediate decline in monthly number of ED-based HIV tests. Disruption of basic health services by the COVID-19 pandemic is a public health concern. Strategies to develop an infrastructure to meet the demands of HIV testing should be implemented to ensure the current HIV prevention during the COVID-19 period.

**Disclosures:**

**All Authors**: No reported disclosures

